# Supplementary material for: Hydrothermal Transformation of Eggshell Calcium Carbonate into Apatite Micro-Nanoparticles: Cytocompatibility and Osteoinductive Properties
Source: Nanomaterials (Basel). 2023 Aug 10;13(16):2299. doi: 10.3390/nano13162299 (PMC10458568; doi:10.3390/nano13162299)
Supplement: Supplementary file 1 [file nanomaterials-13-02299-s001.zip › nanomaterials-2537217-supplementary.pdf]

Supplementary Material

Hydrothermal Transformation of Eggshell Calcium Carbonate into Apatite Micro-Nanoparticles: Cytocompatibility and Osteoinductive Properties

Adriana Torres-Mansilla [1,2], Pedro Álvarez-Lloret [1,*], R. Fernández-Penas [2], Annarita D´Urso [3], Paula Alejandra Baldión [4], Francesca Oltolina [3], Antonia Follenzi [3], Jaime Gómez-Morales [2,*]

[1] Departament of Geology, University of Oviedo, 33005 Oviedo, Spain
[2] Laboratory of Crystallographic Studies, IACT-CSIC-University of Granada, Avda. Las Palmeras, n° 4, 18100 Armilla, Spain
[3] Dipartimento di Scienze della Salute, Università del Piemonte Orientale, "A. Avogadro" Via Solaroli, 17, 28100 Novara, Italy
[4] Departament of Oral Health, Faculty of Dentistry, National University of Colombia, Bogotá 111321, Colombia
* Correspondence: pedroalvarez@uniovi.es (P.Á.-L.); jaime@lec.csic.es (J.G.-M.)

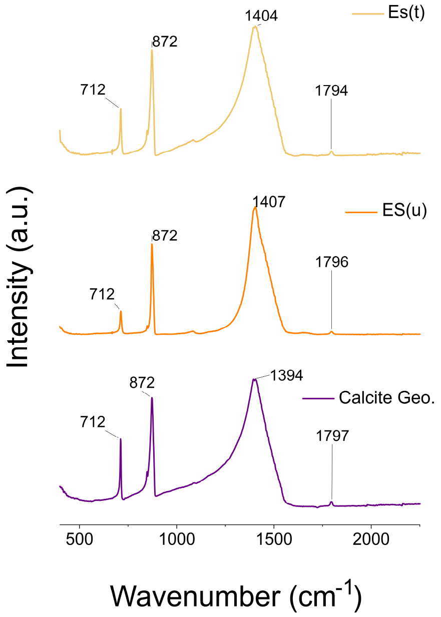

Figure S1. FTIR-ATR spectra of geogenic calcite (bottom), eggshell NaClO-untreated (ES(u) (middle) and NaClO-treated ES(t) (top)

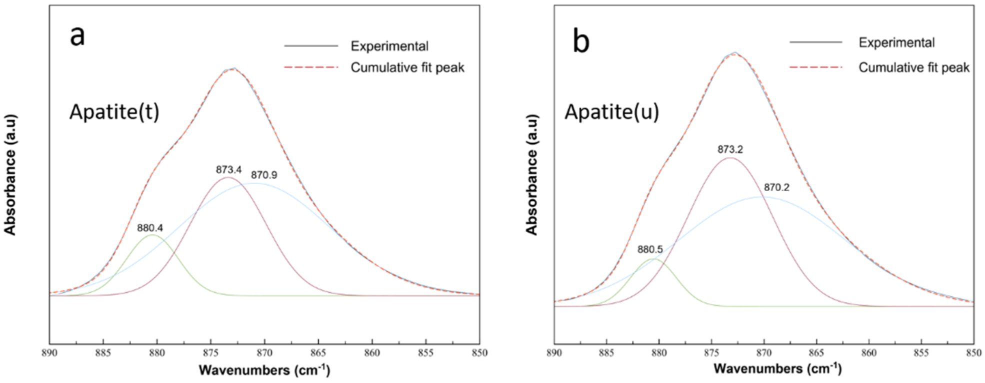

Figure S2. Deconvolution of the low-intensity FTIR absorption band centered at 873 cm^{-1} in samples **(a)** apatite(t)- 200°C and **(b)** apatite(u)-200°C.
